# Clinical aspects and therapeutic approach of drug-induced adverse skin reactions in a quaternary hospital: a retrospective study with 219 cases^☆^

**DOI:** 10.1016/j.abd.2021.06.008

**Published:** 2022-04-02

**Authors:** Júlia Chain Martins, Camila Arai Seque, Adriana Maria Porro

**Affiliations:** a Emergency Medicine, Hospital São Paulo, Universidade Federal de São Paulo, São Paulo, SP, Brazil; b Department of Dermatology, Universidade Federal de São Paulo, São Paulo, SP, Brazil

**Keywords:** Drug eruptions, Drug-related side effects and adverse reactions, Referral and consultation

## Abstract

**Background:**

Adverse drug reactions are frequent, with cutaneous manifestations being the most common. In the hospital environment, the incidence of cutaneous drug reactions varies from 2% to 3%.

**Objective:**

To analyze the profile of cutaneous drug reactions, relating clinical forms, suspected medications, histopathological alterations, systemic repercussions, treatment and course.

**Methods:**

Clinical, retrospective and observational study of patients seen by the Dermatology Interconsultation team from January 2013 to December 2016.

**Results:**

The frequency of cutaneous drug reactions among the evaluated patients was 13.6%, with 219 cases diagnosed. In 65.7%, the reaction was considered mild, of which the most common was exanthema, while in 34.2%, the reaction was considered severe, with DRESS being the main form of reaction(18.2%). Antibiotics (36.5%) and anticonvulsants (10%) were the most involved drugs. In addition to drug discontinuation, systemic corticosteroids were prescribed in 47% of cases and intravenous immunoglobulin (IVIg) in 4.5%. Of the mild forms, in 62%, expectant management and/or exclusive use of symptomatic treatment was used.

**Study limitations:**

Retrospective study, with limitations inherent to this type of investigation; lack of some information in medical records; long evaluation period, with a possible change in external validity.

**Conclusion:**

The most frequently identified clinical form was exanthema, and antibiotics and anticonvulsants were the most frequently involved drug classes. About one-third of the patients had severe cutaneous drug reactions, with DRESS being the main one. Cutaneous drug reactions are frequent in clinical practice, and the dermatologist should be called in as soon as possible to assist in the diagnosis and management of these cases.

## Introduction

According to the World Health Organization (WHO), an adverse drug reaction is defined as any harmful and unintended drug use response that occurs at doses normally used in humans for the prophylaxis, diagnosis, or treatment of diseases.^1^ Adverse cutaneous drug reactions frequently occur, accounting for 10%-15% of all reported cases of adverse drug reactions.^2^ In the hospital setting, the reported incidence ranges from 2% to 3% and varies from mild, self-limiting cases to severe reactions, which can cause significant morbidity and mortality.^3^

In Brazil, few studies have been published on the subject, and only two of them evaluated the prevalence and the clinical forms of cutaneous drug reactions in the hospital environment. In the first study, published in 1984 by Weissbluth et al.,^4^ 12.3% of the patients evaluated by the interconsultation service received a diagnosis of cutaneous drug reactions, while in the second study, published in 2015 by Botelho et al.,^5^ this percentage was 8.2%.

This study aims to analyze the profile of cutaneous drug reactions in patients hospitalized in a quaternary hospital, with the characterization of the demographic profile (gender, age and phototype), clinical forms, suspected medications, histopathological alterations, presence of systemic effects, implemented treatment, and course.

## Methods

This is a clinical, retrospective, observational and descriptive study, which started after approval by the institution’s Research Ethics Committee (protocol number CAAE 59323616.0.0000.5505). All patients attended by the Dermatology Interconsultation service of a quaternary hospital with a diagnosis of cutaneous drug reaction from January 2013 to December 2016 were included, totaling 48 months in the analysis.

The collected sample consisted of 219 patients. Cases in which essential data were incomplete and whose recovery was not possible due to loss of medical records or other reasons were excluded. Data collection was carried out by reviewing the electronic database organized by the Hospital Dermatology Sector of the Institution and by searching the manual and electronic medical records of patients on the Hospital server. When necessary, complementary tests performed during hospitalization and histopathological review of skin biopsies were collected. Photographs were also consulted for better characterization of the clinical form of the cutaneous drug reaction. To define the causes of the cutaneous drug reaction, the drugs used by the patient within a period of up to 14 days before the onset of skin lesions were taken into account.

## Results

### Demographic data

Of the 1607 patients attended by Dermatology interconsultation during the 48-month period, 219 cases were diagnosed with cutaneous drug reaction, corresponding to 13.6%. Of the 219 patients included in the sample, 111 were women (50.6%), and 108 were men (49.6%). The mean age of the attended patients was 47 years, and the median was 49, with a minimum of 1 and a maximum of 95 years. Phototype III was the most prevalent one.

### Inpatient unit

In the analyzed sample, most of the consultations for cutaneous drug reactions (57%) were requested by in-patient units of critically-ill patients, of which 61 (48.8%) came from the emergency room, 42 (33.6%) from intensive care units, 13 (10.4%) from the Kidney and Hypertension Unit and 9 (7.2%) from Transplant Units.

**Figure 1:**
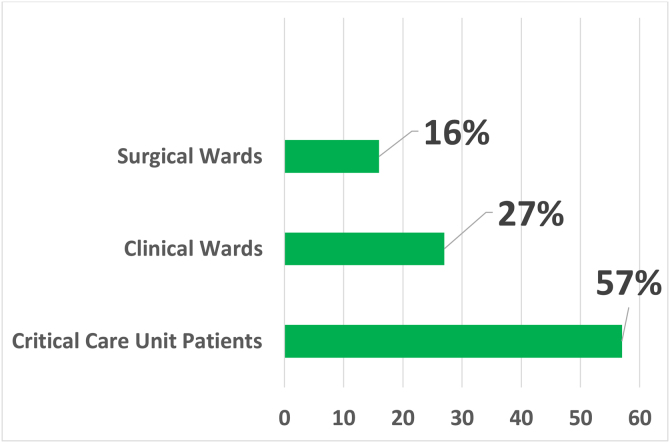
Dermatology Interconsultation Request according to the Hospitalization Unit. Critical Patient Units: Kidney and Hypertension Hospital Unit, Emergency Room, Intensive Care Units and Transplantation Units. Clinical Wards: Internal Medicine, Hematology, Infectious Diseases, Endocrinology, Gastroenterology, Geriatrics, Nephrology, Neurology, Pediatrics, Rheumatology and Supplementary Health Sector Care Unit. Surgical Wards: Neurosurgery, Orthopedics, Urology, Gastrosurgery, Cardiac Surg., Pediatric Surg., Plastic Surg., Vascular Surg., Gynecology, Obstetrics, Ophthalmology and Otorhinolaryngology.

Requests from clinical wards accounted for 26.9% of the total, with most of these being received from the Internal Medicine ward (35.6%). The total number of requests received from surgical wards was 35 (15.9%), and among the specialties, Neurosurgery and Orthopedics were the more involved, with seven requests each (Fig. 1).

### Clinical picture

The time since symptoms onset ranged from one day to seven weeks, and on average, the patients had six days of cutaneous manifestation when the dermatology interconsultation was requested.

As for clinical forms, drug-induced skin reactions were divided into mild (exanthema, erythema multiforme, vasculitis, urticaria, SDRIFE, palmar-plantar erythrodysesthesia) and severe ones (drug reaction with eosinophilia and systemic symptoms - DRESS; Stevens-Johnson syndrome – SJS spectrum/ toxic epidermal necrolysis – TEN; acute generalized exanthematous pustulosis – AGEP),^1^ and in 144 cases (65.7%) the reaction was considered mild, while in 75 (34.2%) it was considered severe. The clinical forms are shown in Table 1.

**Table 1:** Clinical forms of cutaneous drug reactions.

Clinical form	n	%
Mild	144	65.7
Exanthema	111	50.6
Erythema multiforme	14	6.3
Vasculitis	4	1.8
SDRIFE	4	1.8
Urticaria	2	0.9
Others	9	4.1
Severe	75	34.2
DRESS	40	18.2
SSJ/TEN spectrum	22	10.0
AGEP	12	5.4
DRESS + TEN	1	0.4
Total	219	100

SDRIFE, Symmetrical drug-related intertriginous and flexural exanthema; DRESS, Drug reaction with eosinophilia and systemic symptoms; SJS, Stevens-Johnson syndrome; TEN, toxic epidermal necrolysis; AGEP, Acute generalized exanthematous pustulosis.

In the patients with mild reactions, the most frequent clinical form was exanthema, which corresponded to more than 50% of the total sample. Other less prevalent forms were: erythema multiforme (6.3%), symmetrical drug related intertriginous and flexural exanthema (SDRIFE; 1.8%), vasculitis (1.8%), and urticaria (0.9%).

Regarding the severe forms of cutaneous drug reactions, the most prevalent diagnosis was DRESS (40 cases – 18.2%), followed by SJS/TEN spectrum (22 cases – 10%) and AGEP (12 cases – 5.4%). In one case, there was an overlap of DRESS and TEN.

Patients who did not fit in any of the above categories and had mild clinical forms were grouped into “others”, two of which had eczema, three specific reactions to chemotherapy drugs, one dyshidrosiform eruption, one lichenoid eruption, one purpura, and one everolimus-associated ulcer.

### Medications involved

Among the drugs attributed as the cause of cutaneous reactions, antibiotics (36.5%) were the most common: vancomycin (71 cases), meropenem (70 cases), ceftriaxone (36 cases), and polymyxin B (29 cases). Other antibiotics involved with considerable frequency (ten or more cases) were teicoplanin, clindamycin, tazocin, metronidazole, ciprofloxacin, cefepime, amikacin, trimethoprim-sulfamethoxazole, cephalothin, and linezolid. There was an occasional involvement (fewer than ten cases) of oxacillin, amoxicillin + clavulanate, clarithromycin, ampicillin, gentamicin, rifampicin, levofloxacin, azithromycin, penicillin, pyrimethamine, and erythromycin.

The second class of drugs most often associated with cutaneous drug reactions was anticonvulsants (10%), among which phenytoin (26 cases), carbamazepine (9 cases), and phenobarbital (4 cases).

Medications from other classes associated with cases of cutaneous drug reactions were antifungals (fluconazole, amphotericin B and voriconazole), non-steroidal anti-inflammatory drugs (diclofenac, ibuprofen, meloxicam), analgesics (dipyrone, tramadol, morphine), neuroleptics (quetiapine, clonazepam, diazepam, risperidone, haloperidol), chemotherapeutic drugs and others (allopurinol, hydrochlorothiazide, furosemide, amiodarone, everolimus, and oseltamivir), all with sporadic involvement.

The same drug classes showed a similar distribution when the severe forms of cutaneous drug reactions were analyzed separately (Table 2).

**Table 2:** Suspected drugs in adverse drug reactions.

Therapeutic class	All forms of cutaneous drug reaction	%	Severe forms	%
Antibiotics	80	36.5	24	32
Anticonvulsants	22	10.0	16	21.3
Pyrazolone derivatives (dipyrone)	4	1.8	2	2.6
Chemotherapy drugs	4	1.8		
NSAIDs (non-steroidal anti-inflammatory drugs)	3	1.3	1	1.3
Allopurinol			1	1.3
Others	13	5.9	2	2.6
Not identifiable	93	42.3	29	38.6
Total	219	100	75	100

In 93 cases (42.3%), it was not possible to identify the drug responsible for the cutaneous drug reaction, either due to use prior to hospitalization or due to the use of two or more classes of medication temporally correlated with the cutaneous manifestation.

### Complementary exams

Of the total sample of 219 patients, there was an increase in eosinophils in the blood count (reference value above 500 uL) in 115 cases, corresponding to a total of 52.5%. The distribution of cases with eosinophilia was similar between mild (54%) and severe (46%) cutaneous drug reactions. However, the mean level of eosinophils in mild forms was 514.22 uL, while in the severe forms, it was 1925.84 uL. In mild forms, this laboratory alteration was more prevalent in cases of exanthema (87%), while in severe forms, there was a predominance in cases of DRESS (74%), although it also occurred in seven cases of the SJS/TEN spectrum (31.8%) and seven cases of AGEP (58.3%). Considering only the cases of DRESS, the mean eosinophilia was even more important (3267.55/uL), ranging from 654 to 17280 uL.

In 82.6% of the cases, a biopsy of the skin lesions was performed to confirm the diagnosis of cutaneous drug reaction. In mild forms (144 cases), the main histopathological finding was interface dermatitis with inflammatory infiltrate (40% – 58 cases), followed by vasculitis (8% – 12 cases), erythema multiforme (7 cases), and urticarial dermatitis (6 cases). Nonspecific findings were found in 18% (27 cases). The biopsy was not performed in 34 of the 144 patients (23.6%). Among the severe cutaneous drug reactions (75 cases), the most prevalent finding was also interface dermatitis with or without keratinocyte necrosis (81% – 47 cases), followed by findings compatible with AGEP (7 cases), erythema multiforme (2 cases) and urticaria (2 cases). In 13 patients, the histopathological study was nonspecific (17%), and most were DRESS cases (9/13 cases). The examination was not performed in four patients among those who had severe forms.

### Treatment

Regarding the approach adopted by the Dermatology team, the withdrawal of the suspected medication was advised, although this was not always possible, as well as the reduction of the prescription to the minimum necessary in all cases, at the discretion of the attending team.

**Figure 2:**
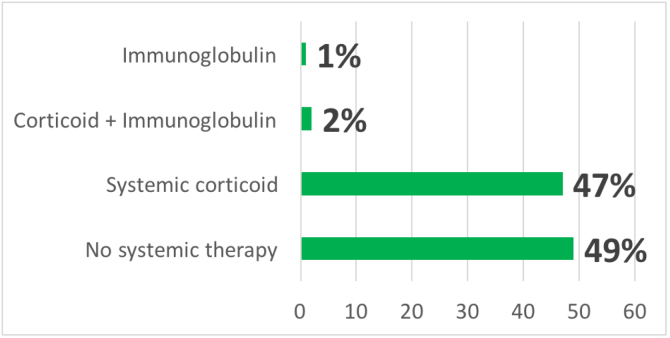
Therapy suggested by the Dermatology Interconsultation team.

In addition to drug withdrawal, systemic corticosteroids were prescribed in 103 cases (47%), intravenous immunoglobulin (IVIg) associated with systemic corticosteroids in five cases (2.2%) and in three cases (1.3%) only IVIg (Fig. 2). Some patients received symptomatic drugs such as antihistamines and/or topical corticosteroids, depending on the clinical form of the cutaneous drug reaction and the patient’s symptoms.

Among the mild forms of cutaneous drug reaction, in most cases (43%), the established approach was an expectant one, comprising only the withdrawal of the suspected medication. The exclusive use of symptomatic medication occurred in 19% of the cases. In 28%, systemic corticosteroid therapy with prednisone at doses lower than 1 mg/kg was carried out, and in 10%, at a dose of 1 mg/kg. There was no indication of IVIg in this group.

In the severe forms, the main course of action was systemic corticosteroid therapy with prednisone at a dose of 1 mg/kg or equivalent (55%). In 11%, systemic corticosteroid therapy with prednisone at doses lower than 1 mg/kg was used. All eight cases that received IVIg in association or not with systemic corticosteroid therapy had severe forms of the SJS/TEM spectrum, and among them, five cases were characterized as TEN. In the group of severe forms, only 24% were treated through expectant conduct or the exclusive use of symptomatic medication.

### Follow up

During the follow up, 163 patients showed improvement or resolution of the condition (74.4%), nine were transferred to the dermatology ward (4.1%), and 14 (6.4%) died.

Among the nine patients who were transferred to the Dermatology ward, all were discharged after clinical improvement and were referred for outpatient follow-up.

Most deaths occurred among the severe cutaneous drug reactions (10 cases), and in four patients, the cause of death was septic shock directly related to a cutaneous infectious focus due to loss of the skin barrier integrity (SJS/TEN) or to complications of IVIg treatment, such as anaphylaxis (one case).

All deaths among the mild forms (4 cases) were due to causes unrelated to the dermatological diagnosis.

## Discussion

Analyzing the demographic data of this study, a slight female predominance (50.6%) was observed, differently from what is evidenced in the most recent literature, whose male to female ratio ranged from 1.14/1 to 1.55/1.^3^, ^6^, ^7^ Regarding the age of the patients, the mean age of 47 years was consistent with the literature data, between 45 and 55 years.^2^, ^3^, ^5^, ^8^^,^^9^

The mean time between symptom onset and the request for interconsultation was six days, very similar to the result of 5.9 days found in a study published by the same group in 2016.^5^ This time interval may reflect the non-identification or non-appreciation of skin changes by the assistant team, which delays the request for dermatological evaluation. It is important to note that the delay in the diagnosis of drug reactions can be fatal, especially in severe forms such as SJS/TEN and DRESS, in which the prognosis is directly related to the early withdrawal of the suspected drug and the implementation of specific treatment when indicated.^10^, ^11^

The class of drugs that most frequently caused cutaneous drug reactions was antibiotics, at 36.5%, followed by anticonvulsants, at 10%, data very similar to those in the literature.^2^, ^3^, ^4^, ^7^, ^8^, ^12^ Among the antibiotics, broad-spectrum drugs (vancomycin, meropenem, polymyxin B) were involved, which are commonly prescribed in a high-complexity quaternary hospital environment and are rarely used in primary and secondary care services, outlining a distinct profile of eliciting drugs compared to non-hospital environments.

The same was observed among severe cutaneous drug reactions (SJS/TEN, DRESS and AGEP), whose main causes were antibiotics (32%) and anticonvulsants (21%), in agreement with a recent study that included 928 cases of the Chinese population, in which the main eliciting drugs were antibiotics (26%), followed by anticonvulsants (21.6%).^13^

However, there are conflicting data in the literature: in studies carried out in the Brazilian population, a statistical association was found between the use of anticonvulsants and severe cutaneous drug reactions.^5^ In these studies, anticonvulsants were still identified as the main agents (40.4%) of severe cutaneous drug reactions, followed by antibiotics (22.8%).^14^ In a study carried out in China in 2008,^12^ the class of anticonvulsants was also the most frequent cause of severe skin reactions, accounting for 38.5% of cases.

Regarding anticonvulsants, it is worth mentioning that the three medications most frequently involved in the present study (phenytoin, carbamazepine, phenobarbital) are aromatic anticonvulsants belonging to the same class. Therefore, in these cases, replacement therapy should be based on a non-aromatic anticonvulsant such as valproic acid or levetiracetam, since changing to a drug of the same class will perpetuate the immunological process triggered in the cutaneous drug reaction.^15^, ^16^

In the present sample, however, it is worth noting that, among the 93 patients (42.3%) in whom it was not possible to identify the eliciting drug, 86 used two or more classes of medication. This probably reflects the complexity of cases admitted to a quaternary hospital.

Regarding the clinical manifestation of the adverse drug reaction, 65.7% of the patients had mild forms, with exanthema being the most common (77% of the mild forms and 50.6% of the total sample). This clinical form of cutaneous drug reaction was also found in other studies in the literature.^4^, ^5^, ^7^, ^8^, ^9^, ^11^, ^17^, ^18^, ^19^ It is noteworthy that there were no cases of fixed drug eruption in the evaluated sample. It is possible that very localized lesions were not observed by the assistant team, which possibly failed to request the Dermatology evaluation.

The severe forms represented 34.3% of the cases, a high frequency when compared to most of the other studies, in which the severe forms represented 3% to 25% of the cases,^2^, ^3^, ^7^, ^8^, ^9^, ^19^ justified by the quaternary profile of high hospital complexity. Among the severe forms, DRESS was the most prevalent one (53.3% of the severe forms and 18.2% of the total), in agreement with a previous study carried out in the same country (45.6% of the severe forms)^14^ and with a study carried out in Malaysia in 2017 (63.6% of severe forms).^3^ These data, however, were different from others found in the literature, in which DRESS significantly showed lower prevalence rates (0.03% to 14.5%).^2^, ^5^, ^6^, ^9^^,^^17^

Histopathological findings in retrospective studies on the frequency and clinical aspects of cutaneous drug reactions have been poorly described. In a series of 25 cases of DRESS, only 7 cases (28%) underwent skin biopsy and histopathological analysis.^20^ This study shows histopathological data from the vast majority of cases (82.6%), the main finding being interface dermatitis, both in mild and severe forms of cutaneous drug reactions. However, in 17% of the patients, this study was nonspecific, and most of them were cases of DRESS.

Among the histopathological findings of cutaneous drug reactions, those related to DRESS raise further discussion in the literature. The presence of atypical lymphocytes in the dermal infiltrate has been associated with the use of anticonvulsants and is most frequently seen in DRESS.^5^ Additionally, the intensity of keratinocyte necrosis, inflammatory infiltrate, and the presence of vasculitis was more related to DRESS compared to exanthema with or without systemic repercussions.^21^ It is postulated that histopathological findings may have prognostic value. The erythema multiforme-like histopathological pattern could indicate greater severity of liver involvement in cases of DRESS.^22^

As therapeutic approach, in addition to the withdrawal of the suspected drug(s) in all cases, systemic corticosteroid therapy was indicated in almost 50% of them. It is important to note that even among severe cutaneous drug reactions, the use of systemic corticosteroids is controversial.

Regarding DRESS, systemic corticosteroid therapy has been considered the main treatment, especially when there is severe involvement of one or more vital organs (liver, kidneys, lungs, heart). Among the 40 cases diagnosed with DRESS, 34 (85%) received systemic corticosteroids, 29 (72.5%) at an immunosuppressive dose (prednisone 1 mg/kg/day or equivalent). In other studies, systemic corticosteroid therapy was prescribed in 34% to 52% of the cases, whereas cases with mild cutaneous and visceral involvement were treated with high-potency topical corticosteroids alone.^20^, ^23^

Among patients in the SJS/TEN spectrum, systemic corticosteroid therapy was given to all patients in the SJS group, and IVIg in association or not with corticosteroids in 58% of the TEN cases, similar to data from a Korean study in which 87% of SJS patients were treated with corticosteroids and 80% of TEN cases with IVIg.^24^

In the literature, the systemic treatment of SJS/TEN remains controversial. Two cohort studies and a review of the literature have not demonstrated any benefit with the use of systemic corticosteroids in the treatment of the SJS/TEN spectrum.^25^, ^26^, ^27^ There is ever increasing evidence on the use of cyclosporine in SJS/TEN, suggesting benefits such as reduced mortality, shorter time required for re-epithelialization, and shorter length of hospital stay.^27^, ^28^, ^29^ In a systematic review published in 2021, which included 67 studies and 2,079 patients, the combined use of corticosteroids and IVIg showed a possible reduction in mortality in patients with the SJS/TEN spectrum.^30^

## Conclusion

The frequency of cutaneous drug reactions among the evaluated patients was 13.6%, with an average of 6 days between the onset of the condition and the request for consultation by the assistant team.

Antibiotics and anticonvulsants were the main classes of drugs involved, and the most frequent clinical form was exanthema. One-third of the cases were considered severe (53.3% DRESS), and there were ten deaths related to the skin condition, which reinforces the role of Dermatology in the monitoring of cutaneous drug reactions in the hospital environment.

The vast majority of cases (82.6%) underwent histopathological analysis of the lesions, confirming the diagnosis and helping to differentiate among the different clinical forms.

Given the important role of the dermatologist in the diagnosis of cutaneous drug reactions, identification of the suspected drug, the differentiation of potentially severe forms, investigation of possible systemic involvement, and implementation of adequate treatment, the ideal situation would have the dermatologist involved as soon as possible in patient evaluation. However, the interval verified in this sample was of almost one week between the onset of the first symptoms and specialized evaluation.

## Financial support

None declared.

## Authors’ contributions

Júlia Chain Martins: Design and planning of the study; collection, analysis and interpretation of data; statistical analysis; drafting of the manuscript; collection, analysis and interpretation of data; critical review of the literature.

Camila Arai Seque: Analysis and interpretation of data; statistical analysis; drafting of the manuscript and critical review of the content; collection, analysis and interpretation of data; participation in research orientation; critical review of the literature; approval of the final version of the manuscript.

Adriana Maria Porro: Design and planning of the study; analysis and interpretation of data; statistical analysis; drafting of the manuscript and critical review of the content; collection, analysis and interpretation of data; participation in research orientation; critical review of the literature; approval of the final version of the manuscript.

## Conflicts of interest

None declared.
